# Direct modulation of TRPC ion channels by Gα proteins

**DOI:** 10.3389/fphys.2024.1362987

**Published:** 2024-02-07

**Authors:** Hana Kang, Jinhyeong Kim, Christine Haewon Park, Byeongseok Jeong, Insuk So

**Affiliations:** ^1^ Department of Physiology, Seoul National University College of Medicine, Seoul, Republic of Korea; ^2^ Department of Physiology, University of California, San Francisco, San Francisco, CA, United States

**Keywords:** G protein, GPCR, Gi pathway, ion channel, TRPC4, TRPC5

## Abstract

GPCR-G_i_ protein pathways are involved in the regulation of vagus muscarinic pathway under physiological conditions and are closely associated with the regulation of internal visceral organs. The muscarinic receptor-operated cationic channel is important in GPCR-G_i_ protein signal transduction as it decreases heart rate and increases GI rhythm frequency. In the SA node of the heart, acetylcholine binds to the M2 receptor and the released Gβγ activates GIRK (I(K,ACh)) channel, inducing a negative chronotropic action. In gastric smooth muscle, there are two muscarinic acetylcholine receptor (mAChR) subtypes, M2 and M3. M2 receptor activates the muscarinic receptor-operated nonselective cationic current (mIcat, NSCC(ACh)) and induces positive chronotropic effect. Meanwhile, M3 receptor induces hydrolysis of PIP_2_ and releases DAG and IP_3_. This IP_3_ increases intracellular Ca^2+^ and then leads to contraction of GI smooth muscles. The activation of mIcat is inhibited by anti-G_i/o_ protein antibodies in GI smooth muscle, indicating the involvement of Gα_i/o_ protein in the activation of mIcat. TRPC4 channel is a molecular candidate for mIcat and can be directly activated by constitutively active Gα_i_^QL^ proteins. TRPC4 and TRPC5 belong to the same subfamily and both are activated by G_i/o_ proteins. Initial studies suggested that the binding sites for G protein exist at the rib helix or the CIRB domain of TRPC4/5 channels. However, recent cryo-EM structure showed that IYY^58-60^ amino acids at ARD of TRPC5 binds with G_i3_ protein. Considering the expression of TRPC4/5 in the brain, the direct G protein activation on TRPC4/5 is important in terms of neurophysiology. TRPC4/5 channels are also suggested as a coincidence detector for G_i_ and G_q_ pathway as G_q_ pathway increases intracellular Ca^2+^ and the increased Ca^2+^ facilitates the activation of TRPC4/5 channels. More complicated situation would occur when GIRK, KCNQ2/3 (I_M_) and TRPC4/5 channels are co-activated by stimulation of muscarinic receptors at the acetylcholine-releasing nerve terminals. This review highlights the effects of GPCR-G_i_ protein pathway, including dopamine, μ-opioid, serotonin, glutamate, GABA, on various oragns, and it emphasizes the importance of considering TRPC4/5 channels as crucial players in the field of neuroscience.

## 1 Introduction

GPCR-G_i_ protein pathways are involved in the regulation of vagus muscarinic pathway under physiological conditions and are closely associated with the regulation of internal visceral organs. The muscarinic receptor-operated cationic channel is important in GPCR-G_i_ protein signal transduction as it decreases heart rate and increases gastrointestinal (GI) rhythm frequency. Among five muscarinic acetylcholine receptors (mAChRs)—M1 to M5—, M2 and M4 receptors primarily utilize G_i/o_ signaling. The M2 receptor, in particular, mediate the effects of parasympathetic stimulation on the heart and GI organs. The most significant involvement among receptor-operated ion channels in this G_i_-related process is definitely that of TRPC channels, especially TRPC4/5 channels.

To begin with, the TRP channel superfamily, comprising 28 mammalian cation channels across seven subfamilies—TRPC, TRPV, TRPA, TRPM, TRPP, TRPN and TRPML (Minke et al., 1975; Zhang et al., 2003; Zhang et al., 2023). Within the subfamilies, TRPC is known to be activated by PLC signaling pathways that lead to membrane depolarization and the elevation in cytosolic Ca^2+^ concentration. Among the various kinds of PLC signaling pathways, the G_q/11_-PLCβ and receptor tyrosine kinase (RTK)-PLCγ pathways are the most commonly known. TRPC ion channels are non-selective cation channels with variable ion selectivity and Ca^2+^ permeability. These receptor-operated channels affects membrane potential and Ca^2+^ signaling in different ways to regulate the physiological conditions (Jeon et al., 2020a). In addition to activation by PLC signaling, direct activation by Gα_i_ is known only for TRPC4/5 channels. Previous studies have shown that Gα_i2_ prefers to bind with TRPC4 whereas Gα_i3_ prefers TRPC5 (Jeon et al., 2008; Jeon et al., 2012). Recently, the dual activation of TRPC4 by both G_i_ and G_q_ signaling pathways has been recognized significant in brain (Yang et al., 2015; Jeon et al., 2020b; Tian et al., 2022). TRPC4 activation requires coincident G_i/o_ stimulation as well as PLC activity (Thakur et al., 2016). Neurons encode distinct messages that reflect the activation of two ion channels, TRPC4 and GIRK, through coincident G_q/11_ and G_i/o_ signaling, transmitting the messages to downstream neurons (Tian et al., 2022).

As cryo-EM structure of TRPC4 and TRPC5 ion channels have been revealed, both channels came out to have similar binding sites−TRPC4/5 activators and inhibitors such as Pico145, Riluzole, HC-070, clemizole, PIP_2_, etc (see also Figure 6)− as their structure significantly overlaps (Duan et al., 2018; Duan et al., 2019; Won et al., 2023). Moreover, several features in intracellular regions of TRPC channels are conserved: the pre-S1 elbow is situated in the N-terminal domain, and the connecting helix runs parallel to the membrane bilayer. However, the binding interface with Gα_i_ protein was conserved only in the N-terminal ankyrin repeat domain (ARD) of TRPC4 and TRPC5 channels, which means both channels may be the only direct modulators for Gα proteins in TRP subfamily (Won et al., 2023). The binding interface of Gα protein with its effector molecules was also found to be conserved in the Gα_i_-bound TRPC5 cryo-EM structure (Lyon et al., 2013; Won et al., 2023). In conjunction with the electrophysiological result demonstrating that Gα_i3_ increases the sensitivity of TRPC5 to phosphatidylinositol 4,5-bisphosphate (PIP_2_), this structural discovery provides evidence that ion channel activity can be directly regulated by Gα protein following GPCR activation. This finding may offer a structural framework for unraveling the crosstalk between two major classes of transmembrane proteins: GPCRs and ion channels. In this review, we specify the G protein related pathway and the direct relationship with TRPC ion channels in various internal organs. Also, possible drug development and disease control studies are introduced by targeting the GPCR-G_i_-TRPC4/5 pathway.

## 2 Two major kinds of G protein: small G protein and heterotrimeric G protein

G proteins, also known as guanine nucleotide-binding proteins, are a family of proteins that act as molecular switches inside cells, and are involved in transmitting signals from a variety of stimuli outside a cell to its interior. The binding and hydrolysis of GTP to GDP, facilitated by specific regulatory factors, govern the activity of these molecules. When in the GTP-bound state, the switch turns on, and, when in the GDP-bound state, the switch turns off. The shutdown of the G protein cascade is possible due to the intrinsic GTPase activity of G proteins, as they belong to the larger group of enzymes called GTPases.

There are two classes of G proteins. The first class functions as monomeric small GTPases (small G proteins), while the second class functions as heterotrimeric G protein complexes. Small G proteins (also known as small GTPases, small GTP binding proteins and Ras protein superfamily) form an independent superfamily within the larger class of regulatory GTP hydrolases. This superfamily is made up of a diverse range of molecules that control a vast number of important processes and possess a common, structurally preserved GTP-binding domain (Agretti et al., 2007). The small G protein superfamily consists of Ras, Rho Rab, Rac, Sarl/Arf and Ran homologs. Within the family of small G proteins, Ras proteins are identified as the best-characterized members. Rasd1 belongs to the Ras superfamily of small GTPase, which is expressed in the brain, heart, liver, kidney, pancreas, skeletal muscle, and placenta (Tu and Wu, 1999; Bernal and Crespo, 2006; Bernal et al., 2021). Activation of Gα_i_ subunits by Rasd1 is known to be the primary mechanism for activating TRPC4 (Wie et al., 2015). Another small G protein that may be a novel target for TRPC5, Rac1, is known to mediate podocyte injury in focal segmental glomerulosclerosis. Studies showed Rac1-activating mutations are responsible for inherited cases of focal segmental glomerulosclerosis, leading to the stimulation of TRPC5 ion channel activity and cytoskeletal remodeling in podocytes (Zhou et al., 2017).

The larger type of G protein, heterotrimeric G proteins are the most commonly found signal transducers in eukaryotic cells, and they mediate the effects of many pharmaceutical products. Heterotrimeric G proteins are the molecular switches that turn on intracellular signaling cascades in response to the activation of GPCRs by extracellular stimuli. GPCRs belong to the largest family of transmembrane receptors and act as the most fundamental signals that are involved in the regulation of internal visceral organs (Kim et al., 2012). Therefore, G proteins have a crucial role in defining the specificity and temporal characteristics of the cellular response (Oldham and Hamm, 2008). The activation of GPCRs promotes an alpha subunit (Gα) of a heterotrimeric G protein to exchange a nucleotide from GDP to GTP inside its pocket, thereby triggering the dissociation of a heterotrimeric G protein (Gαβγ) into Gα and Gβγ. Once activated, Gα proteins amplify the initial signal from the switch by activating effector molecules such as adenylyl cyclase, phospholipase C (PLC), and protein kinases (Liu et al., 2021). Due to the comparable density of ion channels in the plasma membrane (Clapham, 1994), various lines of evidence suggest that not only membrane-bound enzymes but also ion channels could serve as direct effectors of Gα and Gβγ proteins. There is a possibility that ion channels and GPCRs may coexist in close proximity, forming a signaling cluster within a specific region of the plasma membrane (Neves et al., 2002). The recent cryo-EM structure demonstrated that Gα_i3_ could directly activate the TRPC5 channels, and the channel requires both Ca^2+^ and PIP_2_ as essential cofactors for the complete activation of Gα_i3_ (Won et al., 2023).

## 3 The effects of vagus nerve on the visceral and cardiovascular organs

**FIGURE 1:**
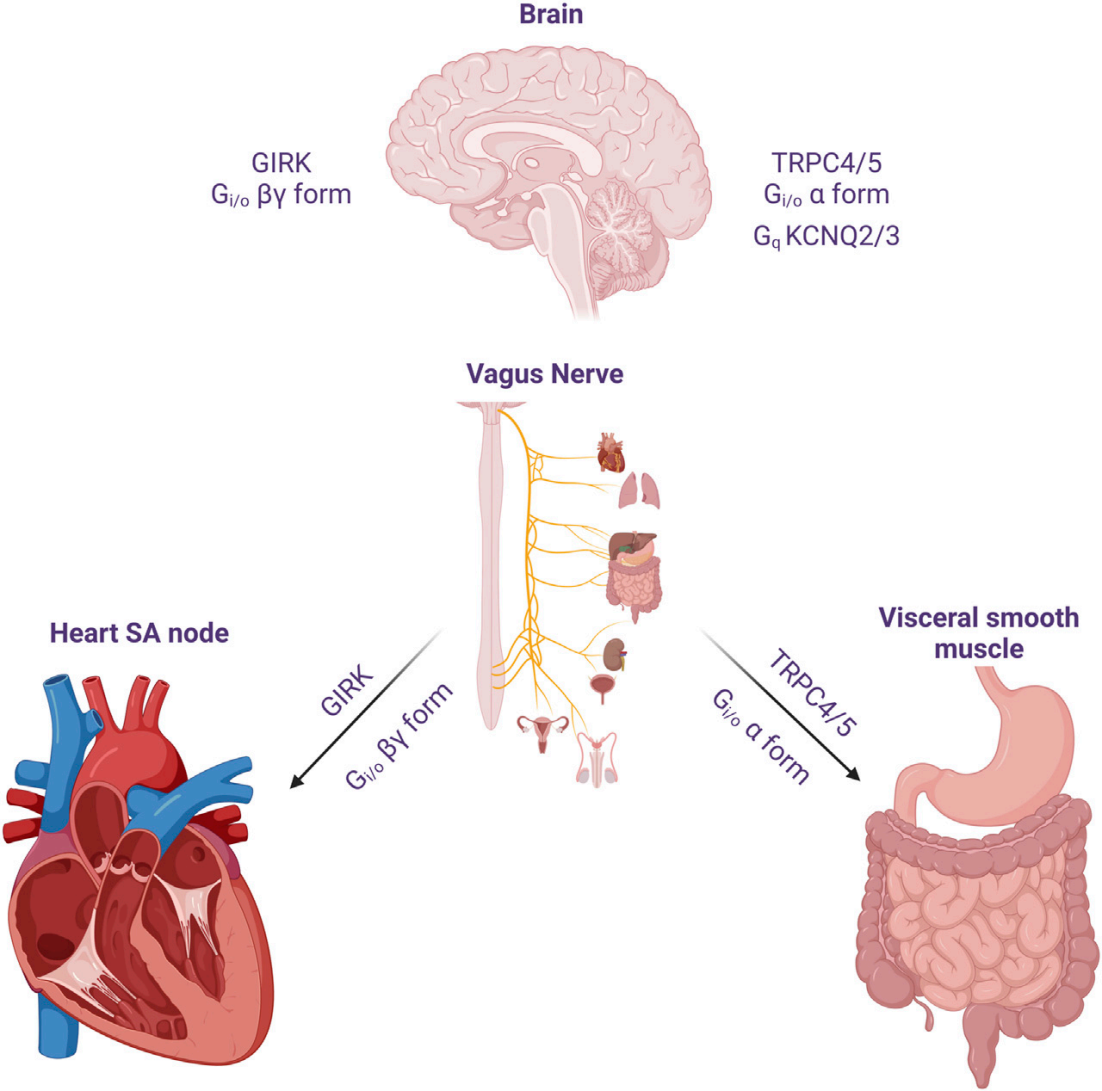
The effect of acetylcholine released from cholinergic neuron on heart, visceral smooth muscles and the brain. The vagus nerve acts on the heart to reduce heart rate and reduce cardiac contractility. One of its important actions is to cause hyperpolarization and reduce heart rate through G_i/o_ protein βγ subunits. In the GI smooth muscle, it activates mIcat cation channels through G_i/o_ protein alpha subunit, causing depolarization and increasing the frequency of pacemaker potential and contraction. In addition, cholinergic nerves increase neuronal excitability by suppressing M current (I_M_) at the superior cervical ganglion sympathetic neurons. Later, the molecular candidate for each ion channels were identified as GIRK(Kir3), TRPC4/5 and KCNQ2/3. An emphasis on the role of TRPC in the G_i_ signaling pathway should be considered in the brain, as well as M channels and GIRK channels.

Neural circuits regulate organ function to stabilize physiological conditions, providing homeostasis to the body’s internal environment (Figure 1). The vagus nerve travels to the internal visceral and cardiovascular organs, where it regulates physiological responses to environmental changes and damages (Rosas-Ballina et al., 2011). ACh released from the vagus nerve binds to the muscarinic receptors. mAChRs comprise a family of five GPCRs, M1 to M5. Three of these receptor subtypes (M1, M3, and M5) have been shown to mainly couple to G proteins of the G_q/11_ family, whereas the remaining two subtypes (M2 and M4) preferentially signal through the G_i/o_ family of G proteins (Hulme et al., 1990). The most well-known example of regulating effects on organs by mAChRs is in the heart, where the activation of M2 receptor results in the activation of Gβγ-dimer, thereby stimulating the GIRK channel to causing membrane hyperpolarization, ultimately slowing pacemaker depolarization (Harvey and Belevych, 2003).

In the GI tract and many other visceral organs, release of ACh from autonomic nerves triggers excitation and contraction of smooth muscle by activating mAChRs. Although various types of mAChRs contribute to concurrent signals for mIcat generation, the activation of M2 receptors predominantly induces the opening of cationic channels. These channels are also subject to modulation by M3 receptors (Bolton and Zholos, 1997; Zholos and Bolton, 1997). Only M2 and M3 receptors mediate contraction in all studied visceral smooth muscles, and M2 receptors contribute to contraction by inhibiting relaxation caused by agents that increase cAMP (Tanahashi et al., 2021). However, some evidences suggest that increase in intracellular Ca^2+^ concentration eliminates the influence of Ca^2+^ release, leading to 1) mIcat inhibition and 2) Gα_o_-regulated depression (Zholos and Bolton, 1997; Yan et al., 2003). In the smooth muscles of various visceral organs, ACh serves as the primary neurotransmitter for excitation (Beech, 1997). It is released from short postganglionic nerves providing parasympathetic innervations to the smooth muscles of organs such as urinary bladder or myometrium (Zholos et al., 2024). The GI tract is equipped with inherent neural plexuses, where ACh is discharged by stimulating motor neurons within the enteric nervous system (Zholos et al., 2024).

Moreover, in the lingual artery, peripheral nerve stimulation resulted in relaxation and membrane hyperpolarization, which inhibitory responses were hindered by atropine (Bevan and Brayden, 1987). ACh plays an important role of endothelium dependent vascular relaxation in the aorta tissue preparation (Freichel et al., 2001). The relaxation was partially blocked in TRPC4 knockout mice (Freichel et al., 2001). On the other hand, Mori group showed that TRPC5 could be nitrosylated by G protein-coupled ATP stimulation in the endothelium. In addition, TRPC1/5 heteromer perform a major role on the NO formation from eNOS in the endothelium via a physical interaction of TRPC5 with eNOS (Yoshida et al., 2006). Interestingly, PKD1 activates TRPC4 in the endothelium through the G_i/o_ protein activation and controls endothelial cell migration and proliferation (Kwak et al., 2018).

### 3.1 Muscarinic stimulation: heart

The signaling of G protein-coupled receptors (GPCR) through G protein-gated inwardly rectifying potassium channels (GIRK) is confined to the cell membrane (Benham et al., 1985; Logothetis et al., 1987). Release of ACh from postganglionic parasympathetic nerve terminals activates muscarinic receptors in the heart. All parts of the mammalian heart are innervated by parasympatheric vagal nerves; vagal activation stimulates the cardiac muscarinic ACh receptors (Capilupi et al., 2020). Stimulation of muscarinic receptors within the heart, specifically the M2 subtype, modulates pacemaker activity and AV conduction, and directly (in atria) or indirectly (in ventricles) effects the force of contraction (Dhein et al., 2001). Mice lacking functional M2 was tested to confirm that M2 subtype is important in the regulation of heart rate as well as anti-nociceptive responses (Gomeza et al., 1999). M1/M3/M5 receptors are also localized in the heart but only M2 are known to mediate significant impacts on heart rate; M2 is the major subtype in cardiac tissue membranes in mammalian heart (Dhein et al., 2001; Willmy-Matthes et al., 2003; Andersson et al., 2011). c-AMP dependent ion channel alteration by M2 muscarinic receptors significantly regulates cardiac function (Harvey and Belevych, 2003). The cardiac GIRK channel, commonly known as Ach-regulated potassium current (I_KACh_), is composed of a heterotetramer comprising GIRK1 and GIRK4 subunits (Luscher and Slesinger, 2010). GIRK channels mediate inhibitory neurotransmission through G protein-coupled receptors (GPCR) in heart and brain; GIRK channels are known to be expressed in the ventricle (Liang et al., 2014). When an agonist binds to GPCR, GDP is substituted to GTP and dissociates Gα and Gβγ (Lambert, 2008). Then, Gβγ activates GIRK channel by binding to its cytoplasmic region.

**FIGURE 2:**
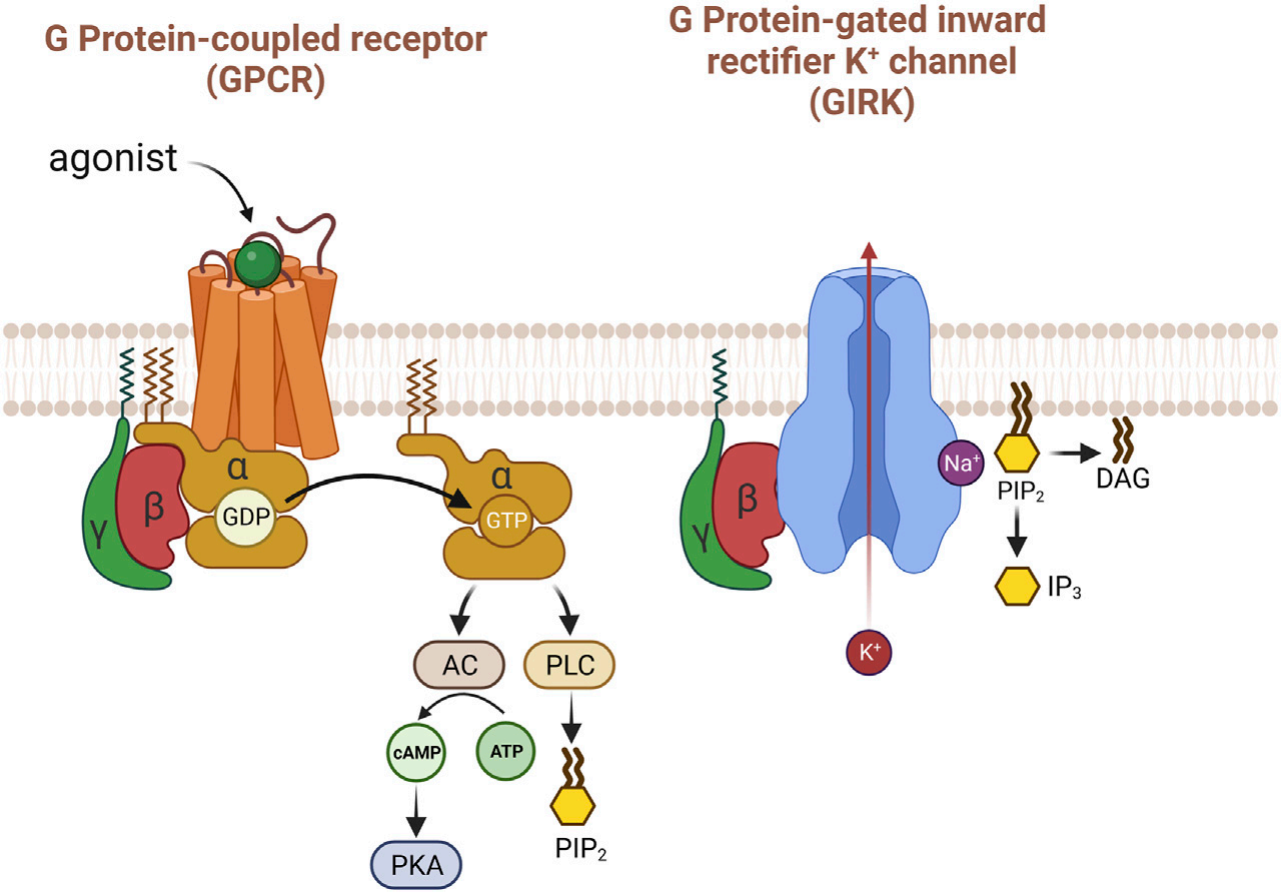
The activation of GIRK with G protein βγ subunits. When acetylcholine binds to the M2 receptor in the heart, several pathways are activated, but the most well-known is that Gβγ mainly and directly activates the GIRK potassium channel. PIP_2_ causes structural changes in GIRK potassium channels to enhance their response to Gβγ. Uniquely, sodium is important for the activity of this potassium channel.

There was a historical controversy regarding which subunits were involved in the activation of GIRK, α or βγ. However, the βγ subunit turned out to be the channel modulator (Logothetis et al., 1987). Decades of years later, the atomic structure of Gβγ-bounded GIRK channel obtained by X-ray crystallography and cryo-EM provided clear insights (Figure 2). The 3.5 Å resolution crystal structure of the mammalian GIRK2 channel in complex with Gβγ protein subunits suggest that the GIRK channel complex with Gβγ differ from the structure without Gβγ, representing the channel state from G protein activation to pre-open conformation and implying the functional pathway from closed to open (Whorton and MacKinnon, 2013). The Gβγ-GIRK interaction sites have mostly been researched in GIRK1 and GIRK2 (Yokogawa et al., 2011). Gβγ protein binds to multiple contact sites in the complex with GIRK. Several studies based on mutagenesis suggest that extra amino acid residues within Gβ might be involved in the regulation of basal or induced activities in GIRK (Albsoul-Younes et al., 2001; Zhao et al., 2003). Unlike Gβγ, there is still no crystal structures of GIRK-Gα and the interaction between the two are determined as GDP-bound, that is considered inactive. When the GIRK channel is open, the rate of membrane depolarization slows down due to the hyperpolarization of membrane potential.

Recent study demonstrates that neurons generate specific signals, that are produced by activating TRPC4 and GIRK channels, reflecting concurrent stimulation of G_q/11_ and G_i/o_ pathways. The simultaneous transmission of neurotransmitters via the G_q/11_ and G_i/o_ pathways is translated into distinct electrical responses through the collaborative functions of TRPC4 and GIRK, facilitating communication to downstream neurons (Tian et al., 2022). On the other hand, Gβγ subunits are barely involved in the direct activation of TRPC4 or TRPC5 by Gα_i_ unlike GIRK channels. PIP_2_ has been identified as a regulator of the gating of GIRK channel, and GIRK’s x-ray crystal structure of GIRK revealed that each channel interacts with four PIP_2_ molecules. Additionally, the interaction between the TRPC4 channel and PIP_2_ is well-established, emphasizing the crucial role of PIP_2_ in maintaining these channels (Kim et al., 2012). In addition, As PIP_2_ has been recognized to regulate membrane-associated proteins and act as a signal molecule in phospholipase C-linked G_q_-coupled receptor (GqPCR) pathways and GqPCR-induced inhibition of ion channels by means of PIP_2_ depletion occurs in a receptor-specific manner (Cho et al., 2005a; Cho et al., 2005b).

### 3.2 Muscarinic stimulation: GI smooth muscle

The cholinergic GI smooth muscle contraction is regarded as an M3 response mediated by the Ca^2+^ signaling pathway, which includes G_q/11_-coupled activation of phospholipase C-β (PLC-β) (So and Kim, 2003). PLC cleaves the membrane lipid phosphatidylinositol 4,5-bisphosphate (PIP_2_) into the second messengers diacylglycerol (DAG) and inositol 1,4,5-trisphosphate (IP_3_), leading to Ca^2+^ release. As the M3/G_q_/PLCβ pathway is ubiquitous in the GI smooth muscle, the DAG-dependent mechanism might as well contribute to mIcat activation in guinea-pig ileum and stomach and mouse ileum (Unno et al., 2006).

In all types of visceral smooth muscles, ACh serves as the primary excitatory neurotransmitter. It is released from short postganglionic nerves providing parasympathetic innervation to the smooth muscles of visceral organs. Over time, research on all types of visceral smooth muscle has dramatically increased as work on GI Smooth muscle increased concurrently. Smooth muscle researches established direct correlation between membrane depolarization, action potential frequency and the force of ACh -induced contractions (Bulbring, 1954). The effects ascribed to non-selective increase of membrane permeability to Na^+^, K^+^, and Ca^2+^, but not Cl^−^. Thus, patch clamp technique was used to directly record and characterize mIcat (muscarinic cation current) as a nonselective, voltage-sensitive cation current that switches on by ACh stimulation on single smooth muscle cells of the rabbit jejunum in 1985 (Benham et al., 1985). After this first publication of directly recorded patch clamp data was published, numerous investigations revealed the role of a pertussis-toxin sensitive G protein (Inoue and Isenberg, 1990a; Komori and Bolton, 1990; Komori et al., 1992; Zholos et al., 1994; Zholos et al., 2004) and intracellular Ca^2+^ on mIcat potentiation (Inoue and Isenberg, 1990b; Komori et al., 1993). The activation of mIcat is inhibited by anti-G_i/o_ protein antibodies in GI smooth muscle (Kim et al., 1998a; Yan et al., 2003), indicating the involvement of G_i/o_ protein in the activation of mIcat.

**FIGURE 3:**
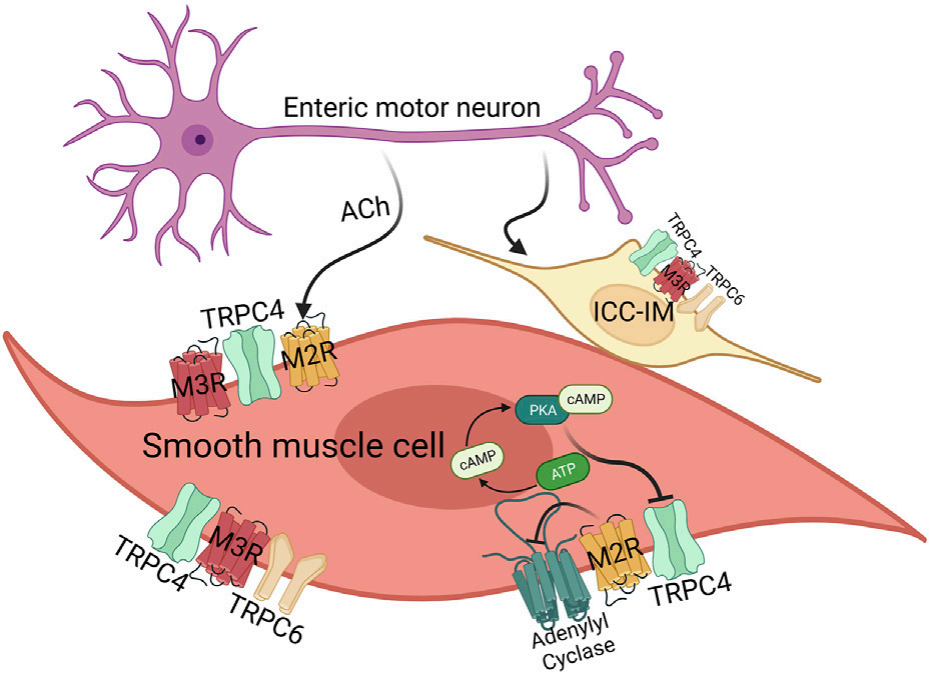
The effects of acetylcholine on smooth muscle cells and ICC-IM via M2/3 receptors. GI smooth muscle mainly expresses M2 (80%) and M3 (20%) receptor, and ICC-IM expresses M3 receptor. In case of TRPC channels, smooth muscles mainly express TRPC4 and TRPC6. Both M2/3 receptor are important for TRPC4 function via G_i_ and G_q_ proteins, whereas M3 receptor activates TRPC4 and TRPC6 via the G_q_-PLC-DAG pathway. M2 receptor alone activates TRPC, but in this case, it also works through G_i_-AC-cAMP-PKA as well as G_i_ protein itself.

These initial discoveries have indicated a mutual reliance of mIcat on the activation of both M2R and M3R. As mentioned earlier, M2R couples to pertussis-toxin sensitive G_i/o_ proteins and M3R is coupled to phospholipase C(PLC)/IP_3_ pathway of G_q/11_ proteins (Figure 3). The concurrent oscillations of intracellular Ca^2+^ concentration and mIcat activation disclosed the PLC/IP_3_ pathway to IP_3_-induced Ca^2+^ release, which was observed in single guinea-pig ileal smooth muscle cells (Komori et al., 1993; Zholos et al., 1994). Such potentiation of mIcat during peaks of IP_3_-induced Ca^2+^ release enhances membrane depolarization, reaching the action potential threshold and causing voltage-dependent Ca^2+^ entry via voltage gated Ca^2+^ channel. When combined with a concurrent peak of IP_3_-induced Ca^2+^ release, it elicits smooth muscle contraction. The fact is, the change in intracellular Ca^2+^ concentration induced by L-type Ca^2+^ channel is remarkably higher than the changes induced by muscarinic receptor-operated cation channels (Kim et al., 1998b). Synthetic smooth muscle cells within the vascular system reduce the expression of L-type voltage-gated Ca^2+^ channels while simultaneously elevating the expression of low voltage-activated Ca^2+^ channels and TRPC channels (House et al., 2008). TRPC4/5 and TRPC6 have been known to be related to the Ca^2+^ responsive pathways that play a role in the transcriptional regulation (Freichel et al., 2001; Tiruppathi et al., 2002; Kuwahara et al., 2006). In addition, TRPC4/6 have been suggested to have a role in the *in vivo* regulation of GI motility by influencing the contraction of smooth muscle cells (Tsvilovskyy et al., 2009), producing the nonselective cationic currents through muscarinic receptor stimulation in intestine smooth muscle cells (Figure 3).

Since interstitial cells of Cajal (ICCs) play a crucial role in cholinergic neurotransmission within visceral smooth muscles, these cells can be considered as an additional target contributing to smooth muscle complications following general anesthesia (Zholos et al., 2024). There was a study of the transcriptome in ICCs uncovered the presence of 550 ion channel isoforms in jejunal and colonic ICCs (Figure 3). This includes channels that have been previously identified as responsive to general anesthetics in various cell types (Lee et al., 2017). Notably, mouse intestinal ICCs express TRPC4 and TRPC5 channels. Experimental evidence using the specific TRPC4/5 blocker ML204 and the direct agonist EA has highlighted the significance of these channels in modulating spontaneous intracellular Ca^2+^ oscillations and pacemaker activity (Lee et al., 2020). ICCs serve as the pacemaker cells that initiate and propagate electrical slow waves in the GI smooth muscles. Although the pacemaker activity originates from Ano-1 or TRPM7, TRPC channels induce depolarization after eating and increase the frequency of the pacemaker activity (Figure 3). Along with TRPC4, TRPC6 have also been identified in ICCs in the same preparation (Epperson et al., 2000; Lee et al., 2017).

TRPC5 is expressed in a variety of smooth muscle cell types and TRPC4 has been demonstrated to exhibit broad expression within the endothelial tissue, suggesting its potential role in orchestrating the regulation of vascular smooth muscle through endothelium-dependent mechanisms (Freichel et al., 2001; Tiruppathi et al., 2002). TRPC4 came out to be the most important TRPC channel regarding the smooth muscle cells as they have been found in a widespread of smooth muscle cells from different vascular beds and has response to ACh triggered muscarinic receptor activation in smooth muscle cells of the GI tract. The ACh -activated TRPC channels would result in the depolarization of smooth muscle cells in the intestine, leading to subsequent activation of L-type Ca^2+^ channels and inducing contraction (Tsvilovskyy et al., 2009). The impact of muscarinic effects on numerous channels poses the complex challenge of discerning their respective significance, particularly within the interactions involving M2 and M3 receptors. Nonetheless, the activation of mIcat undeniably stands out as a primary mechanism for exciting GI smooth muscle (Zholos, 2006).

Many studies have suggested that the enteric nervous system plays an important role in normal GI smooth muscle development (Chamley-Campbell et al., 1979; Langer et al., 1994; McHugh, 1995). The bidirectional communications between the evolving enteric nervous system and GI smooth muscle seem to have a crucial impact on the regular differentiation, maturation, and functioning of both tissue types. The significance of specific receptor ligand pathways in regulating these essential cell-to-cell interactions throughout GI development has been confirmed, which may lead to clinical importance of certain GI diseases and disorders (McHugh, 1995).

## 4 TRPC4/5 activation mechanism: PIP_2_, Ca^2+^, and Gα

TRPC4/5 channel is a molecular candidate for mIcat (Zhu et al., 2003; Lee et al., 2005) and can be directly activated by constitutively active Gαi^QL^ proteins. TRPC4 and TRPC5 belong to the same subfamily and both are activated by G_i/o_ proteins. G_i2_ prefers to bind with TRPC4 whereas G_i3_ prefers TRPC5 (Jeon et al., 2008; Jeon et al., 2012). Initial studies suggested that the binding sites for G protein exist at the rib helix of TRPC4/5 channels or the CIRB domain (Jeon et al., 2012). However, recent cryo-EM structure showed that IYY^58-60^ amino acids at ARD bind with G_i3_ protein (Won et al., 2023). Main debate concerns with the role of PIP_2_. We showed that PIP_2_ is essential for maintaining TRPC5 channel activity. Recently, we directly applied PIP_2_ with inside-out patch mode and activated TRPC5 channels. When the binding sites was mutated, the mutants did not respond to intracellularly applied PIP_2_. The role of Gα_i_ protein was to enhance the affinity of TRPC5 channels to PIP_2_ at the physiological PIP_2_ range (Won et al., 2023). Other research groups showed that PIP_2_ inhibited TRPC5 tonically at the basal level, and depletion of PIP_2_ decreased the activation time constant and rapidly increased the TRPC5 current (Thakur et al., 2016). Furthermore, Gudermann group suggest that DAG is a real activator for TRPC4 and TRPC5 channels because PIP_2_ depletion cause TRPC4/5 to respond to DAG (Storch et al., 2017). Another important point is the roles of PLCδ1. We showed that PLCδ1 was activated by Ca^2+^ influx through TRPC4 and played a negative role on TRPC4 currents (Ko et al., 2023). Zhu group showed the contrary results. They needed G_i_ protein and PLCδ1 to activate TRPC4 channels (Thakur et al., 2016). When PLCδ1 was inhibited, TRPC4 was not activated by agonists, even in the presence of G_i_ proteins. Ca^2+^ and H^+^ ion were suggested as activators (Jeon et al., 2020a; Thakur et al., 2020). The exact roles of PIP_2_ would be revealed when the cryo-EM structure of the PIP_2_-bounded TRPC5 channel is obtained.

**FIGURE 4:**
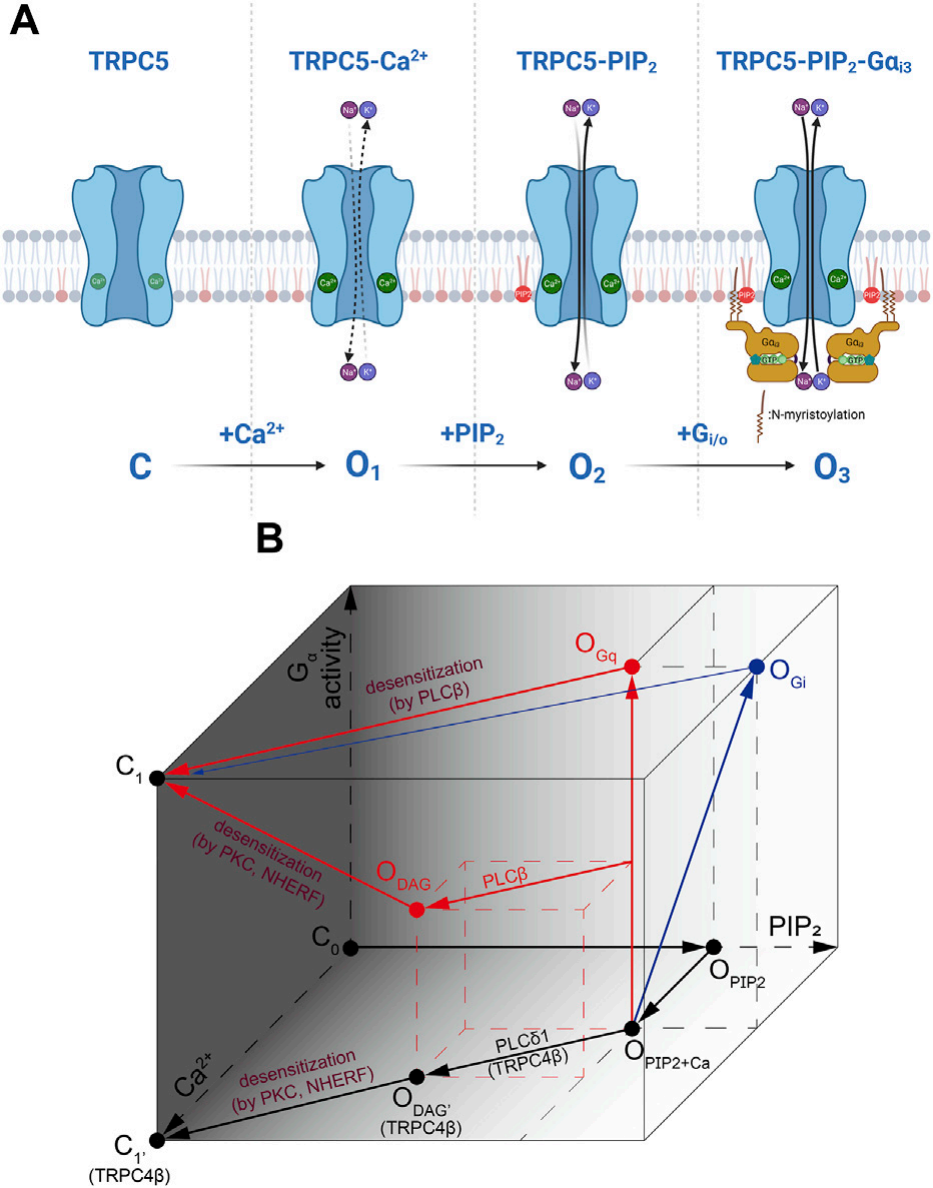
The activation process of TRPC4/5 via Ca^2+^, PIP_2_ and Gα. **(A)** During the inside-out patch clamp recordings, we induced initial TRPC5 activity with Ca^2+^ and subsequently activated the TRPC5 current by applying Gα_i3_ protein or PIP_2_. Gα_i_ and/or Gα_q_ proteins are considered to directly induce further activation of the TRPC5 channel. However, in actual physiological situations, it might be assumed that PIP_2_ always tends to be attached to the TRPC5 ion channel. **(B)** A cube-shaped schematic diagram describing the overall gating mechanism of TRPC4/5 channels. Three axes represent PIP_2_ (X-axis) or Ca^2+^ (Y-axis) binding with the channels, and the strength and/or progress of the Gα activity (Z-axis). Red and blue arrows represent sequences mediated by G_q_ and G_i_, respectively.

Recent cryo-EM structure supports the fact that PIP_2_ binding site on TRPC5 is located near the S2-S3 linker, S4-S5 linker, TRP helix, and helix-loop-helix region. As Gα_i_ protein binds to TRPC5, increase in PIP_2_ affinity leads to the increase as well. This means that Gα_i_ protein is not necessary to open the TRPC5 channel, but the intracellular Ca^2+^ concentration and PIP_2_ affinity (or binding) may be the direct trigger for opening the TRPC5 channel. Both Ca^2+^ and PIP_2_ have the potential to serve as a cofactor in the activation of the channel at the intracellular leaflet, consistent with findings from previous studies (Ningoo et al., 2021). Full activation of Gα_i3_ to the channel may require the involvement of all three factors: Ca^2+^, PIP_2_, and Gα_i3_ (Figure 4). Also, PLCδ1 does not bind to TRPC5 unlike it does with TRPC4, which causes TRPC5 to have basal current with a high concentration of PIP_2_.

We summarizes the complex interaction of G protein, DAG, PIP_2_ and calcium as in Figure 4. First, to indicate that the channel is not open without PIP_2_, the side consisting of the Gα and Ca^2+^ axes is darkened and points on the side set to be closed (C_0_, C_1_). In the presence of PIP_2_ and Ca^2+^, channels are partially open (O_PIP2+Ca_). As Gα_i_ activity increases and Gα_i_ bind directly to channels (O_PIP2+Ca_→O_Gi_), the PIP_2_ sensitivity of the channel increases. In the diagram, this change is depicted by the increase in PIP_2_ concentration, although it does not imply the actual elevation in PIP_2_ concentration. G_q_ activity also opens TRPC4/5 channels potently (O_PIP2+Ca_→O_Gq_). Both open states induced by G_q_ and G_i_ reach to closed state through activation of PLCβ (O_Gq_ or O_Gi_→C_1_). However, the transition from G_i_-open state is not powerful, as depicted. This process is accompanied by an increase in Ca^2+^ and a decrease in PIP_2_. TRPC4/5 channels can open by under specific conditions when diacylglycerol (DAG) is generated from PIP_2_ molecule (O_DAG_). PIP_2_ hydrolysis occurs in TRPC4β by PLCδ1. At this time, PLCδ1 becomes active due to an increase in Ca^2+^ independent of any Gα activities. Therefore, the process is drawn at the bottom (O_PIP2+Ca_→O_DAG’_). Since PLCβ is activated by G_q_, it is plotted diagonally to reflect the Ca^2+^ increase and PIP_2_ depletion at the time point in which G_q_ activity has progressed to some extent along the vetical O_PIP2+Ca_–O_Gq_ line (middle of O_PIP2+Ca_–O_Gq_ line→O_DAG_). DAG-induced open states become closed when the C-terminus of the channel is phosphorylated by PKC, followed by binding with PDZ motif of Na^+^/H^+^ exchanger regulatory factor (NHERF). The process occurs concurrently with the advancement of G_q_ activity, depletion in PIP_2_, and an increase in Ca^2+^ levels, reaching the dark side mentioned first and entering a closed state (O_DAG_→C_1_). But Gα activity is not needed in the case of TRP4β (O_DAG’_→C_1_).

## 5 GPCR-G_i/o_-TRPC4/5 signal pathway in neuron

**FIGURE 5:**
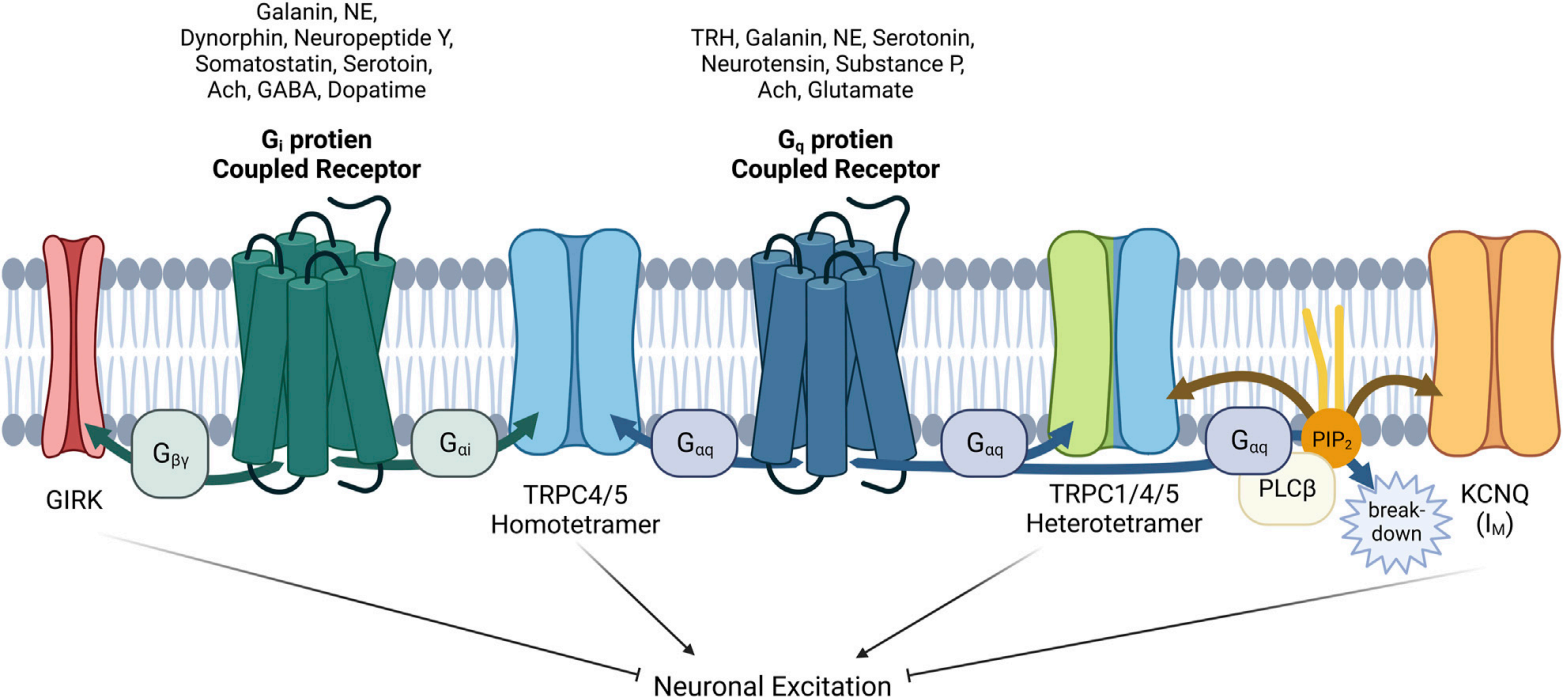
Complex interactions among GIRK, TRPC1/4/5 and KCNQ channels with GPCRs via G proteins α or βγ subunits and PIP_2_ in neurons. The role of GPCRs in neurons is intricate and complicated. When acetylcholine acts on neuronal cells, the physiological function of at least three ion channels (TRPC1/4/5, GIRK, KCNQ) must be analyzed considering their distribution and expression. In addition, βγ subunit also inhibits Ca_V_ channels, contrary to activation of GIRK. As for TRPC1/4/5, heteromers seems to play a major role in the brain recently, so heteromers should always be considered together as well as homomers. In addition to acetylcholine, galanin, norepinephrine (NE), and serotonin must always be considered as a neurotransmitter acting on both G_i_ and G_q_ proteins. Furthermore we must remember that GABA, dopamine, somatostatin, neuropeptide Y, and dynorphin, which act on G_i_-coupled GPCR pathway, can work inducing the direct binding of Gα_i_ proteins to TRPC4/5 homomer. In our hands, Gα_q_ also binds directly (Myeong et al., 2018), but the structure of Gαq bound TRPC4/5 has not yet been revealed. GPCRs that suppress the M current have been demonstrated to utilize G_q/11_ proteins for the activation of phospholipase C, leading to the hydrolysis of PIP_2_. PIP_2_ serves as a diffusible second messenger within the membrane, directly influencing the activity of KCNQ currents.

GPCR-Gi/o protein signaling pathway in neurons is an essential component of the complex network of signaling mechanisms that regulate neuronal function (Figure 5). Recent studies indicated the direct relationship between neurological disorders and TRPC4/5 channels. Increased TRPC5 S-glutathionylation by oxidative stress contribute to neuronal damage in striatum that may result in Huntington’s disease (Hong et al., 2015). Dysfunction in TRPC4 may lead to epilepsy or autism spectrum disorder (Zheng, 2017; Gupta et al., 2023; Zhou et al., 2023). Freichel group showed that heteromeric TRPC1/4/5 channels are involved in depression and anxiety (Broker-Lai et al., 2017; Chu et al., 2020) TRPC1/4/5 channels play a role in the development of morphine tolerance and hyperalgesia. Prolonged exposure to morphine results in an increase in the expression of TRPC1/4/5 channels in the spinal cord (Chu et al., 2020). TRPC1/4/5 channels also possess developmental functions in neurons. TRPC5 regulates hippocampal neurite development (Greka et al., 2003), and dendrite patterning (Puram et al., 2011). TRPC4 in rat dorsal root ganglion neurons are known to be necessary for neurite outgrowth. Suppression of TRPC4 immuno-reactivity resulted in decrease in the length of neurites in cultured dorsal root ganglion neurons, confirming the necessity of TRPC4. Nerve injury causes increase in TRPC4 as well (Wu et al., 2008). Later research reported activation of TRPC4β, TRPC4 splice variants, through Gα_i_ regulates the morphogenesis of dendrites in cultured hippocampal neurons (Jeon et al., 2013).

TRPC4 as well as TRPC1 support the repetitive neural spiking in brain, confirming the various functions on the neuronal pathway. Relatively high expression level of TRPC4 in lateral septum promotes firing rate. Lateral septum receives signals from various brain regions, extending from hippocampus to amygdala, where diverse neurotransmitters such as ACh, dopamine, glutamate, GABA, and serotonin converge. The depolarization of plateau potential, responsive to electrical stimulation in the presence of blockers for inotropic GABA and glutamate receptors, were shown to be mediated by the G_q/11_-coupled group 1 metabotropic glutamate receptors (Gallagher et al., 1995). Later, Zhu group showed that both G_i_- and G_q_-coupled signaling pathways are important for the spike firing in lateral septal nucleus and the response differs from G_q_-only or G_i_-only signaling (Yang et al., 2015; Jeon et al., 2020b; Tian et al., 2022). Two interconvertible depolarization responses (below-threshold-depolarization and above-plateu-depolarization) of TRPC4-group 1 metabotropic glutamate receptor activation contribute to patterns in lateral septal neuron firing activities (Phelan et al., 2012; Tian et al., 2014; Phelan et al., 2023). Another research has shown that not only TRPC4 but also TRPC1 are essential for an intrinsic membrane conductance mediating the plateau potential in lateral septal neurons (Phelan et al., 2012).

Flockerzi group elaborately showed that heteromeric TRPC1/4/5 channels are the major functional channels in the brain using multiple specific antibodies for TRPC1/4/5, multi-epitope affinity purifications, and high resolution liquid mass spectrometry (nano-LC-MS/MS) (Kollewe et al., 2022). The amount of TRPC proteins determined in each sample by nano-LC-MS/MS were finally combined to deduce the abundance of each isoform in all possible tetrameric configurations. The importance of hetero-tetramers is rising, given that only minor portions of the TRPC1, TRPC4, and TRPC5 proteins in the brain are present in homomers (13%, 6%, 9%, respectively). The majority is incorporated into three categories of heteromers: TRPC1/C4, TRPC1/C5, and TRPC1/C4/C5 (Kollewe et al., 2022). These findings are notable since homo- or hetero-tetramers modify channel properties significantly, such as Ca^2+^ permeability, PIP_2_ sensitivity, and I-V curve. Further studies of the heteromeric TRPC1/4/5 channels in GPCR signaling pathway are necessary to understand their activity under physiological conditions.

Shapiro group put an emphasis on the role of TRPC in the G_i_ signaling pathway in the brain, as well as M channels and GIRK channels (Carver and Shapiro, 2019; Carver et al., 2020; Carver et al., 2021). Sohn group also suggested that TRPC5 mediates the effects of leptin and serotonin via POMC neurons (Gao et al., 2017), and this effect is independent of altering GIRK channel activity (Sohn et al., 2011). Recently, G_i/o_-coupled GPCR in the paraventricular nucleus of the hypothalamus was found to antagonize the anorexic effect of serotonin agents via K_ATP_ channels (Yoo et al., 2021). Melanocortin 4 receptors (MC_4_Rs) in parasympathetic preganglionic neurons activate K_ATP_ channels via G_s_ signaling, but in sympathetic preganglionic neurons, they activate putative nonselective cation channels (Sohn et al., 2013). In case of this neuronal circuit regulating feeding behavior and energy metabolism (involving POMC or NPY/AgRP neurons), TRPC5 channels are more crucial than GIRK and Ca_V_ channels. Most importantly and recently, Zhu group suggested that the lateral septal nucleus utilizes a minimum of two channels, TRPC4 and GIRK, both of which are modulated by G_i/o_ and G_q/11_ pathways. While the G_i/o_ and G_q/11_ pathways compete in their effects on GIRK, they cooperate in producing a self-propagating all-or-none activation of TRPC4. Zhu group emphasized that these nonlinear interactions allow for the encoding of coincident signaling, particularly the relative degrees to which the 2 G protein pathways are being activated, resulting in discernible action potential firing patterns (Tian et al., 2022).

In case of TRPC5, it exhibits the highest expression in the brain, mostly in CA1 pyramidal cell, amygdala, cingulate gyrus, and cerebellar nuclei (Riccio et al., 2002). M2R and M4R, the G_i/o_-coupled GPCRs, are localized to both presynaptic and postsynaptic terminals, where they inhibit neuronal excitation with the coupled-G_i/o_ proteins. G_i/o_-coupled GPCRs mediate inhibitory signals. For example, activation of these receptors can lead to a decrease in cAMP levels, which, in turn, can modulate ion channel activity and neurotransmitter release. This inhibition is crucial for maintaining the balance of excitatory and inhibitory signals in various processes in nervous system, such as synaptic transmission and neuronal excitability. Knockout mice of M4R, not M2R, show increased basal ACh release in the hippocampus (Tzavara et al., 2003). Considering with the recent finding that TRPC5 is the direct effector of G_i/o_ protein triggered by GPCR activation (Won et al., 2023), researchers should particularly consider the novel GPCR-Gα_i_-TRPC5 pathway, especially in studies related to neuronal diseases.

## 6 TRPC4/5 drug discovery

TRP channels are known to be transducers of exogenous and endogenous noxious cues. The last decade has been superb with dramatically high resolution of molecular structures that have allowed us to learn the molecular intricacies of TRP channels using cryogenic electron microscopy. These findings, in combination with functional studies, have provided insights into the role played by these channels in the generation and maintenance of pain (Rosenbaum et al., 2022). The expression pattern in brain nuclei of TRPC4 and TRPC5 also show possibilities to become a novel TRP targets involved in pain processing. While the emphasis on the generation of pain has traditionally been centered on sensory neurons, there is a high possibility of discovering new drugs based on non-neuronal cell types, which can also impact pain perception (Rosenbaum et al., 2022). Research on TRPC4 knockout rats showed tolerance to visceral pain responses, whereas somatic pain responses were uninfluenced (Koivisto et al., 2022). In addition, the non-selective TRPC4/5 antagonist, 4-methyl-2-(1-piperidinyl)quinoline (ML-204), inhibited visceral pain responses in wild-type rats, confirming the role of TRPC4 in visceral pain. The application of ML-204 to amygdala results in suppression of mechanical hypersensitivity and attenuated neuropathic pain behavior in rats with spared nerve injury (Wei et al., 2015). Another TRPC4/5 antagonist HC-070 developed by Hydra and Boehringer Ingelheim is currently in clinical trial for the treatment of anxiety disorder and depression (Wulff et al., 2019). HC-070 also had a significant anti-hypersensitivity effect in the established phase of the chronic constriction injury model (Jalava et al., 2023). TRPC5 inhibitor GFB-887, currently in phase 2 clinical trial, is being developed by Goldfinch Bio for the treatment of kidney disease. GFB-887 was first developed as a treatment for diabetic nephropathy but GFB-887 showed the best result in patients with focal segmental glomerulosclerosis, a rare kidney disease marked by blood vessel scarring in the glomerulus (NCT number: NCT04387448). These findings suggest that centrally mediated TRPC4 and TRPC5 antagonists could relieve visceral and neuropathic pain (Blum et al., 2019). On the other hand, TRPC4 and TRPC5 activator Englerin A has been developed for cancer therapy as it can inhibit growth of tumor cell lines at nanomolar concentrations (Carson et al., 2015). Englerin A is a selective inhibitor of renal cancer cell growth compared to normal kidney cells and cancer cell lines of different origin (Akbulut et al., 2015). Selectivity turns out to be one of the most important factors in drug development, meaning that a discovery of a precise structure of Englerin A binding site in TRPC4/5 would be crucial for further research (Jeong et al., 2019; Kim et al., 2019).

**FIGURE 6:**
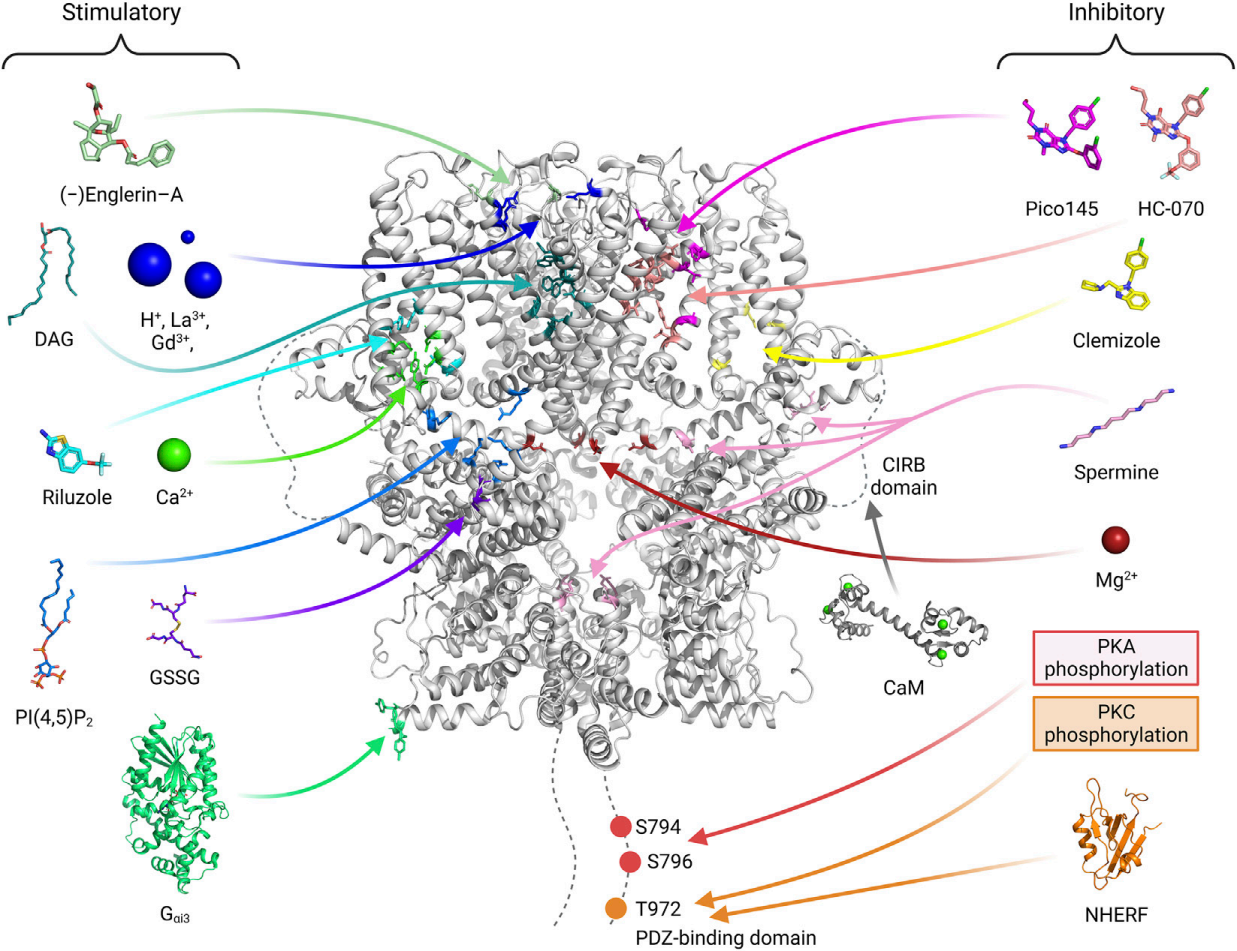
The binding sites of activators or inhibitors on the TRPC4/5 channels. The binding or regulating sites of various substances that modulate the channel activity are shown with a human TRPC5 channel structure (PDB ID: 7X6I). Stimulatory molecules or atoms are placed on the left side, and their binding sites are depicted with residues of blue-toned color. On the right side, inhibitory molecules, atoms, or modifications are illustrated, and their binding sites are indicated with red-toned color. Given that the effect of calmodulin (CaM) varies depending on the research group (Kim et al., 2006; Vinayagam et al., 2020), the binding site of the molecule is indicated with a gray color. In drug development, effective drugs can be developed by using computers to predict binding to various sites and then verifying these predictions through experiments. The drugs made so far are concentrated in the membrane area. The GSSG glutathionylation site will be a good target considering the recent results showing the relation of TRPC5 R175C mutation and impaired intellectual ability (Leitao et al., 2022). It is also connected to zinc, which in turn connected to redox sensing and zinc poisoning. For each substance, references are added. DAG (storch et al., 2016; Song et al., 2021; Wright et al., 2020; PDB ID: 7E4T), H^+^ ion (Semtner et al., 2007), La^3+^, Gd^3+^ (Jung et al., 2003), Gα_i_, PIP_2_ (Won et al., 2023; PDB ID: 7X6I), (−)-Englerin A (Jung et al., 2003; Kim et al., 2020), Riluzole (Yang et al., 2022; PDB ID: 7WDB), GSSG (Hong et al., 2015), Ca^2+^ (Duan et al., 2018; Duan et al., 2019; Vinayagam et al., 2020; Song et al., 2021; Won et al., 2023) are stimulatory. Pico145 (Wright et al., 2020; PDB ID: 6YSN), HC-070, Clemizole (Song et al., 2021; PDB ID: 7D4Q, 7D4P), spermine (Kim et al., 2016; 2020), Mg^2+^ (Obukhov and Nowycky, 2005), PKA (Sung et al., 2011), PKC (Zhu et al., 2005) and NHERF (Storch et al., 2017; Otsuguro et al., 2008; PDB ID of PDZ domain: 1G04) are inhibitory.

Recent TRPC4/5 inhibitors block both TRPC4/5 with relatively similar potency, which confirms that they are not ready for pharmaceutical use (Zheng, 2022). The zinc binding site and CIRB site in the more variable cytosolic domain may also be promising for developing drugs that can differentiate TRPC4 and TRPC5 (Zheng, 2022). Furthermore, the PIP2 binding site may be a novel site to target as well. Activators or inhibitors for TRPC4/5 can be classified into three types, extracellular type, transmembrane (TM) type and cytosolic type (Figure 6). Ions like H^+^ ion, La^3+^ or Gd^3+^, binds to extracellular sites and activates TRPC4/5 channels (Jung et al., 2003; Semtner et al., 2007). This sites might be suitable for drugs which are water soluble and have charges. Many drugs, like riluzole, pico145, HC-070 or clemizole, binds to TM area (Wright et al., 2020; Song et al., 2021; Yang et al., 2022). Physiological modulators like Ca2^+^, DAG, or PIP_2_ also binds to TM domain. The posttranslational modification, like PKA or PKC phosphorylation and glutathionylation, occurs on cytosolic area. We showed that G protein binds to cytosolic ARD and activates TRPC5 channels. For specific effect of drugs, multiple sites should be considered like G protein binding and PKA phosphorylation sites, PIP_2_ and NHERF binding sites, or DAG binding and PKC phosphorylation sites (Zhu et al., 2005; Sung et al., 2011; Storch et al., 2017). Focusing only on the TM sites might not be enough for visualizing specific effect of drugs on TRPC4/5. Recent genetic study showed that R175C gain of function mutation in TRPC5 cause an impaired intellectual ability (Leitao et al., 2022). In this case, the drug affecting glutathionylation might improve said symptoms.

## 7 Conclusion

Ion channels, especially TRPC channels are now considered as novel target to be directly regulated by Gα_i_ proteins. GPCR-G_i_ protein pathways are involved in the regulation of vagus muscarinic pathway under physiological conditions and are closely associated with the regulation of internal visceral organs. The direct and indirect modulations of TRPC channel by G protein play an important role in the muscarinic stimulation that is known to involve the GPCR-G_i_ protein pathway including dopamine, μ-opioid, serotonin, glutamate, GABA, and the complex interaction between GIRK and TRPC4/5 should be considered in the field of neuroscience. However, two big questions need to be further addressed: the structure and functional involvement of heteromeric TRPC channels and the PIP_2_ binding site regarding the TRPC channel and G protein complex. Heteromeric TRPC channels are naturally expressed at relatively high levels in the brain, which may be a key for a drug development in the field.
